# Promoting N_2_ electroreduction to ammonia by fluorine-terminating Ti_3_C_2_T_x_ MXene

**DOI:** 10.1186/s40580-021-00264-9

**Published:** 2021-05-10

**Authors:** Yu Ding, Junbo Zhang, Anxiang Guan, Qihao Wang, Si Li, Abdullah M. Al-Enizi, Linping Qian, Lijuan Zhang, Gengfeng Zheng

**Affiliations:** 1 Laboratory of Advanced Materials, Department of Chemistry, Faculty of Chemistry and Materials Science, Fudan University, Shanghai, 200438 China; 2 Department of Chemistry, College of Science, King Saud University, Riyadh, 11451 Saudi Arabia

**Keywords:** MXene, Surface functionalization, Electrocatalysis, N_2_ reduction reaction, Fluorine

## Abstract

Two-dimensional MXene-based materials are potential of presenting unique catalytic performances of electrocatalytic reactions. The surface functionalization of MXene-based catalysts is attractive for developing efficient electrocatalysts toward nitrogen reduction reaction. Herein, we reported a Ti_3_C_2_T_x_ MXene with a medium density of surface functionalized fluorine terminal groups, as an excellent N_2_ reduction reaction electrocatalyst with enhanced adsorption and activation of N_2_. The Ti_3_C_2_T_x_ MXene catalyst showed a production rate of ammonia as 2.81 × 10^–5^ μmol·s^−1^·cm^−2^, corresponding to a partial current density of 18.3 μA·cm^−2^ and a Faradic efficiency of 7.4% at − 0.7 V versus reversible hydrogen electrode in aqueous solutions at ambient conditions, substantially exceeding similar Ti_3_C_2_T_x_ MXene catalysts but with higher or lower densities of surface fluorine terminal groups. Our work suggests the capability of developing surface functionalization toolkit for enhancing electrochemical catalytic activities of two-dimensional MXene-based materials.

**Supplementary Information:**

The online version contains supplementary material available at 10.1186/s40580-021-00264-9.

## Introduction

Artificial nitrogen fixation to ammonia (NH_3_) plays a critical role in fabricating agricultural fertilizers and maintaining the earth’s ecosystems [1–3]. The traditional NH_3_ synthesis in industry depends heavily on the Haber–Bosch process with high temperatures of 350–550 ℃ and pressures of 150–350 atm [4, 5]. In recent years, new strategies, such as biological [6], photocatalytic [7] and electrocatalytic [8–10] approaches, have been reported for ammonia synthesis. In particular, electrocatalytic nitrogen reduction reaction (N_2_RR) can use water as hydrogen source and proceed in ambient conditions, suggesting an attractive feature of clean ammonia production with low carbon footprint [11]. Nevertheless, the development of N_2_RR has been largely limited by its low current densities, limited Faradaic efficiency (FE) values, and slow NH_3_ production rates, which are ascribed to the large reaction energy barriers during NH_3_ adsorption and activation processes [12]. It is critical to design robust electrocatalysts that can efficiently adsorb, activate and convert N_2_ into NH_3_.

Two-dimensional (2D) materials, such as graphene [13], metal–organic frameworks [14], black phosphorus [15], have been drawing great attention of researchers for N_2_RR, owning to their unique 2D structures and unconventional chemical properties [16]. MXenes, one of the novel 2D materials synthesized by selective etching of the aluminum layers from the precursor MAX phases [17], have been demonstrated with applications in supercapacitors [18], batteries [19], and electrochemical N_2_RR [20–22]. For instance, Luo et al. [23] reported that Ti_3_C_2_T_x_ MXene on stainless steel mesh functioned as efficient N_2_RR electrocatalysts with a FE of 5.78%. On the other hand, the terminal groups (T_x_), mainly oxygen (O)-containing or fluorine (F)-terminations, can be tuned to affect the electrocatalytic performances of Ti_3_C_2_T_x_ MXene [24]. Previously, density functional theory (DFT) calculations suggested that Ti_3_C_2_ MXene with O-containing terminal groups can combine N_2_ more strongly than that with F-terminal groups [25]. The bond length of N_2_ for Ti_3_C_2_ MXene with F-terminal groups was calculated to be slightly larger than that with O-containing terminal groups [26]. The computational calculations also indicated that a moderate proportion of F-termination on Ti_3_C_2_T_x_ MXene was theoretically beneficial to the adsorption and electrocatalytic activation of nitrogen. Thus, efforts are needed to develop efficient and tunable functionalization on the surface of Ti_3_C_2_T_x_ MXene to optimize its N_2_RR performance and achieve highly selective NH_3_ production.

Herein, we developed the surface modification of MXene-based catalysts to optimize the N_2_RR performance. The fluorine-terminal groups on the MXene surface were modified by treating with different concentrations of fluorine-containing acids and subsequent alkalization. Due to the combined effects including hydrogenation and N_2_ activation, Ti_3_C_2_T_x_ MXene with a medium F-terminal group density (designated as Ti_3_C_2_T_x_-medium F) exhibited the optimal electrocatalytic N_2_RR performances. The NH_3_ production rate was 2.81 × 10^–5^ μmol·s^−1^·cm^−2^ in 0.01 M Na_2_SO_4_ electrolyte at − 0.7 V versus reversible hydrogen electrode in ambient conditions, corresponding to a FE of 7.4% and a partial current density up to 18.3 μA·cm^−2^.

## Experimental

Ti_3_C_2_T_x_ MXene nanosheets were synthesized via a fluorine-containing etching method [27, 28], which selectively etched the Al layers in Ti_3_AlC_2_ with fluorine-containing acid, followed by exfoliating steps to obtain Ti_3_C_2_T_x_ MXene nanosheets. To modify the density of fluorine terminal groups on the surface of Ti_3_C_2_T_x_ MXene, different fluorine-containing acids were used. In brief, 2 g of LiF was dissolved in 20 mL of 6 M HCl solution, and stirred for 5 min to form a high fluorine-containing acid environment to prepare the Ti_3_C_2_T_x_ MXene with high surface density of F-terminal groups; while 20 mL of 10% hydrofluoride acid (HF) was used to obtain a medium surface density of F-terminal groups. In order to further reduce the fluorine ratio of the terminal groups on the MXene surface, Ti_3_C_2_T_x_ MXene with medium density of fluorine-terminal groups was immersed into 0.5 M KOH solution to replace fluorine terminal groups by hydroxyl groups.

In our experiments, 1 g of Ti_3_AlC_2_ MAX precursor was slowly added into the aforementioned solutions respectively, and the obtained mixture was stirred for 24 h at room temperature to achieve different surface functionalization of fluorine or hydroxyl groups. Afterwards, the resulting solution was washed with deionized (DI) water and centrifuged at 3500 rpm for several times until pH of the solution was 6‒7. To prepare few-layer Ti_3_C_2_T_x_ MXene nanosheets, the sediment was then dispersed in DI water and ultrasonicated for 30 min in ice bath with Ar gas bubbling. The few-layer Ti_3_C_2_T_x_ MXene solution was then obtained by centrifugation at 3500 rpm for 30 min. A polyvinyl difluoride (PVDF) membrane was utilized to filter Ti_3_C_2_T_x_ MXene product and the obtained powder was dried with membrane for 12 h at room temperature.

## Results and discussion

**Fig. 1:**
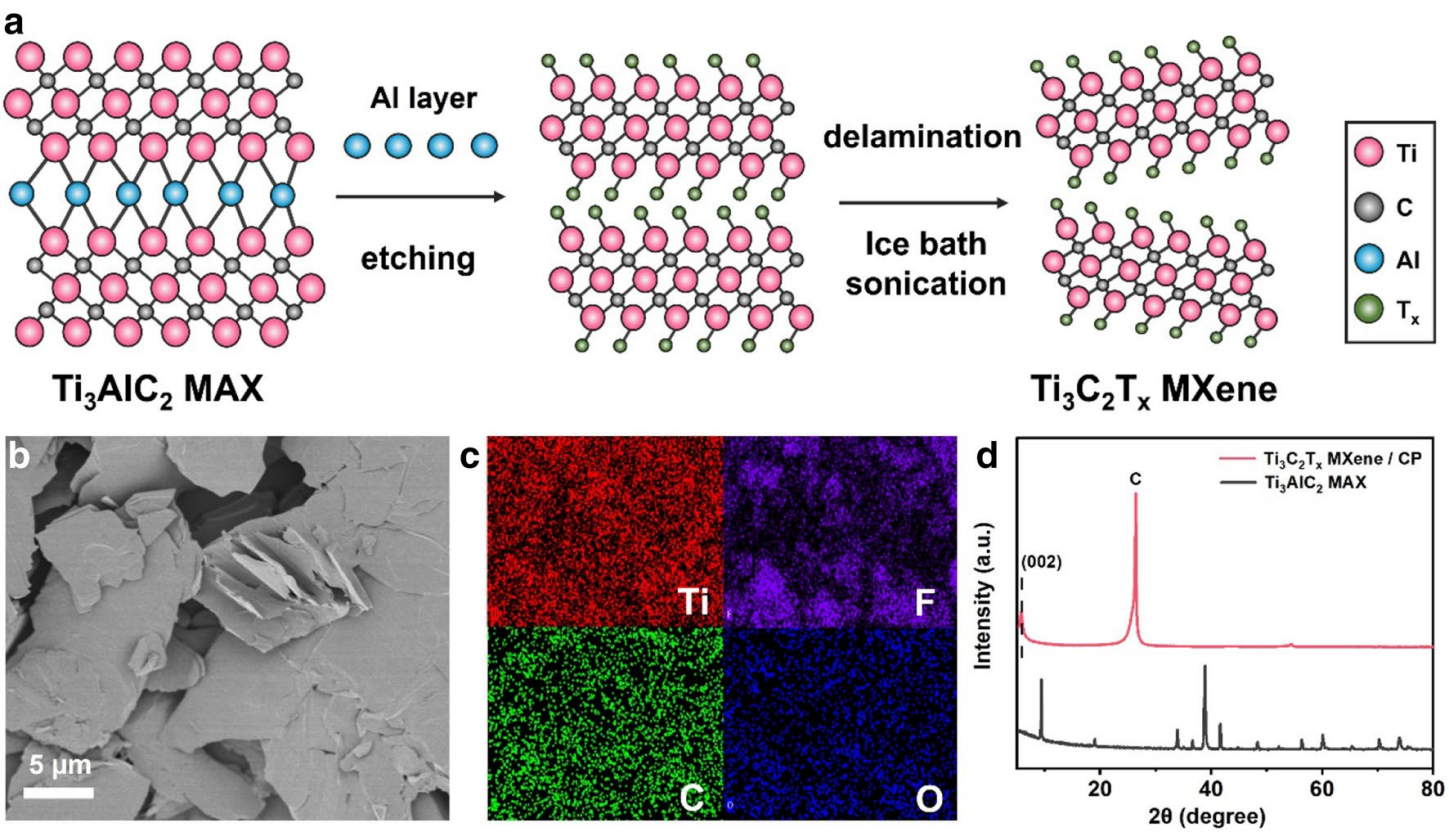
**a** Schematic of the synthesis procedure of the Ti_3_C_2_T_x_ MXene. **b** SEM images of Ti_3_C_2_T_x_ MXene. **c** EDX elemental mapping profiles of Ti_3_C_2_T_x_ MXene with Ti (red), F (purple), C (green), and O (blue) distributions. **d** XRD spectra of Ti_3_C_2_T_x_ MXene loaded on carbon paper (CP)

The synthesis process of Ti_3_C_2_T_x_ MXene nanosheets included two successive steps [27, 28]: selective etching of aluminum (Al) layer on MAX phases with a fluorine-containing etching method, and delaminating of MXene layers by sonication (Fig. 1a). After these two steps of preparation, the original structure of Ti_3_AlC_2_ MAX phase (Additional file 1: Fig. S1) was transformed into few-layer, 2-dimensional Ti_3_C_2_T_x_ MXene (Fig. 1b). The surface of Ti_3_C_2_T_x_ MXene was covered by different terminal groups, mainly including O-containing groups (e.g.–O, − OH) and fluorine groups [29]. Energy-dispersive X-ray spectroscopy (EDS) elemental mapping analysis showed that Ti, C, O, and F elements were evenly distributed on the obtained Ti_3_C_2_T_x_ MXene (Fig. 1c). The color of few-layer Ti_3_C_2_T_x_ MXene appeared as a blackish green color instead of pure black (Additional file 1: Fig. S2a), and the Tyndall scattering effect was also observed (Additional file 1: Fig. S2b). These two empirical phenomena indicated the formation of few-layer, sheet-like structure of Ti_3_C_2_T_x_ MXene.

The X-ray powder diffraction (XRD) pattern exhibited the characteristic peaks of Ti_3_C_2_T_x_ MXene, which differed greatly from the Ti_3_AlC_2_ MAX precursor (Fig. 1d). After etching, the peak (2θ ≈ 9.28°) on Ti_3_AlC_2_ MAX phase exhibited a negative shift to a low angle (2θ ≈ 5.89°), corresponding to the characteristic lattice (002) plane of Ti_3_C_2_T_x_ MXene [30]. No additional peaks were observed for Ti_3_C_2_T_x_ MXene except for the peaks of carbon paper. The differences of the XRD patterns between Ti_3_C_2_T_x_ MXene and Ti_3_AlC_2_ MAX indicated the successful etching of Al layers from Ti_3_AlC_2_ MAX phases.

Scanning electron microscopy (SEM) images showed that all the synthesized Ti_3_C_2_T_x_ MXene were 2D sheet-like structures (Additional file 1: Fig. S3). The ratio of F-terminal groups to all the surface groups of Ti_3_C_2_T_x_ MXene was measured by EDS profiles (Additional file 1: Fig. S4), which was calculated as 82% (designated as Ti_3_C_2_T_x_-high F, indicating that it was etched by a high-concentration fluorinated acid) and 48% (designated as Ti_3_C_2_T_x_-medium F, indicating that it was etched by a medium-concentration fluorinated acid). To further decrease the ratio of fluorine groups on the surface of MXene, an alkalization treatment was conducted to Ti_3_C_2_T_x_-medium F MXene (Experimental section), and the fluorine proportion was reduced to 24% (designated as Ti_3_C_2_T_x_-low F).

**Fig. 2:**
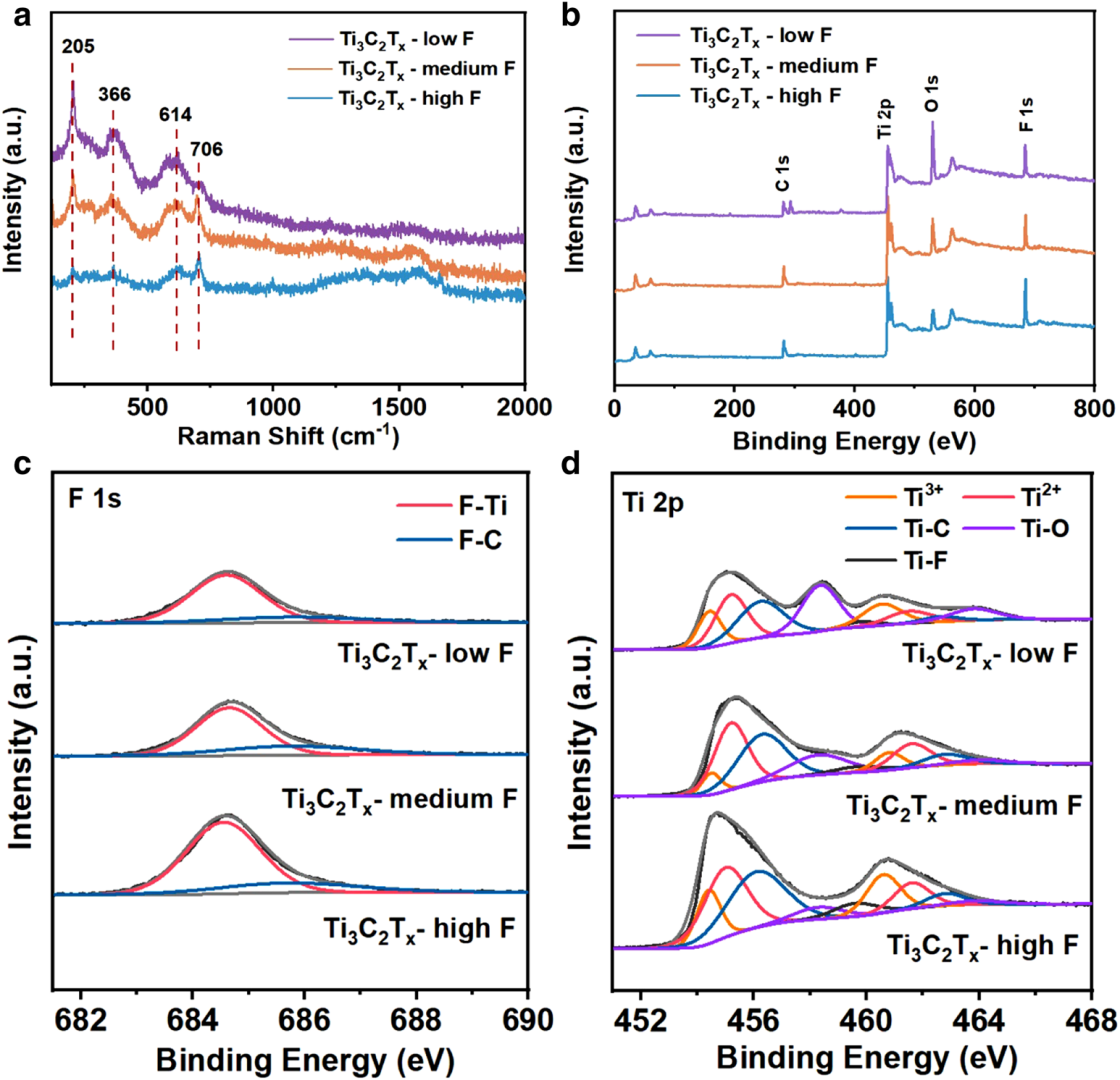
**a** Raman spectra and **b** XPS spectra of Ti_3_C_2_T_x_-low F, Ti_3_C_2_T_x_-medium F and Ti_3_C_2_T_x_-high F. **c** F 1 s spectra, and **d** Ti 2p XPS spectra of Ti_3_C_2_T_x_-low F, Ti_3_C_2_T_x_-medium F and Ti_3_C_2_T_x_-high F MXene samples

To investigate the structures of Ti_3_C_2_T_x_ MXene with different amounts of fluoride, Raman and X-ray photoelectron spectroscopy (XPS) were conducted to qualitatively investigate the fluorine ratio in all the terminal groups. As shown in the Raman spectra (Fig. 2a), the relative intensity of vibrational modes for Ti_3_C_2_T_x_ indicated the densities of terminal groups on MXene. The Raman shifts at 205 and 366 cm^−1^ were respectively associated to the A_1g_ and E_g_ vibration modes of the C–Ti–O structure. The Raman peak at 614 cm^−1^ corresponded to the C–Ti–OH structure [23], and the Raman peak at 706 cm^−1^ represented the A_1g_ vibrations of carbon. The relatively large intensity of the characteristic Raman peaks at 205, 614, and 366 cm^−1^ corresponded to the O-containing terminal groups, and suggested that the densities of O-terminal groups were in the order of Ti_3_C_2_T_x_-low F > Ti_3_C_2_T_x_-medium F > Ti_3_C_2_T_x_-high F. This trend also confirmed that the densities of F-terminal groups followed the order as: Ti_3_C_2_T_x_-high F > Ti_3_C_2_T_x_-medium F > Ti_3_C_2_T_x_-low F.

The survey XPS spectra of all the Ti_3_C_2_T_x_ MXene samples with different densities of fluorine terminal groups exhibited same element peaks (Fig. 2b), which assigned to F 1 s, O 1 s, Ti 2p and C 1 s, respectively. Among these samples, Ti_3_C_2_T_x_ MXene-high F showed the highest intensity of F 1 s and the lowest intensity of O 1 s, indicating the highest ratio of fluorine terminal groups among these samples, in good accord with the EDX result. The F 1 s XPS spectra presented two main peaks at 684.5 and 685.9 eV (Fig. 2c), corresponding to the F–Ti and F–C bonds, respectively. For the O 1 s spectra (Additional file 1: Fig. S5), the peaks at 529.3, 531.2 and 533.4 eV were ascribed to C–Ti–O, C–Ti–OH species, and the adsorbed H_2_O on the MXene surface. With the increased concentrations of alkali solutions, the density of C–Ti–O species on the MXene surface increased and the fluorine terminal groups decreased. For the Ti 2p XPS spectrum (Fig. 2d), four doublets were fitted to indicate the valance and bond structures of Ti. The peaks centered at 454.4 and 460.6 eV referred to Ti^3+^; the peaks at 455.0 and 461.7 eV were assigned to Ti^2+^; the peaks at 456.2 and 462.9 eV were associated with the Ti−C bond; the peaks at 458.4 and 464.3 eV were ascribed to the Ti−O bond; and the peak at 459.5 eV was attributed to the Ti−F bond. The relative intensities of Ti−O and Ti−F bonds also indicated that the fluorine terminal group density on the MXene surface followed the order of Ti_3_C_2_T_x_-high F > Ti_3_C_2_T_x_-medium F > Ti_3_C_2_T_x_-low F.

**Fig. 3:**
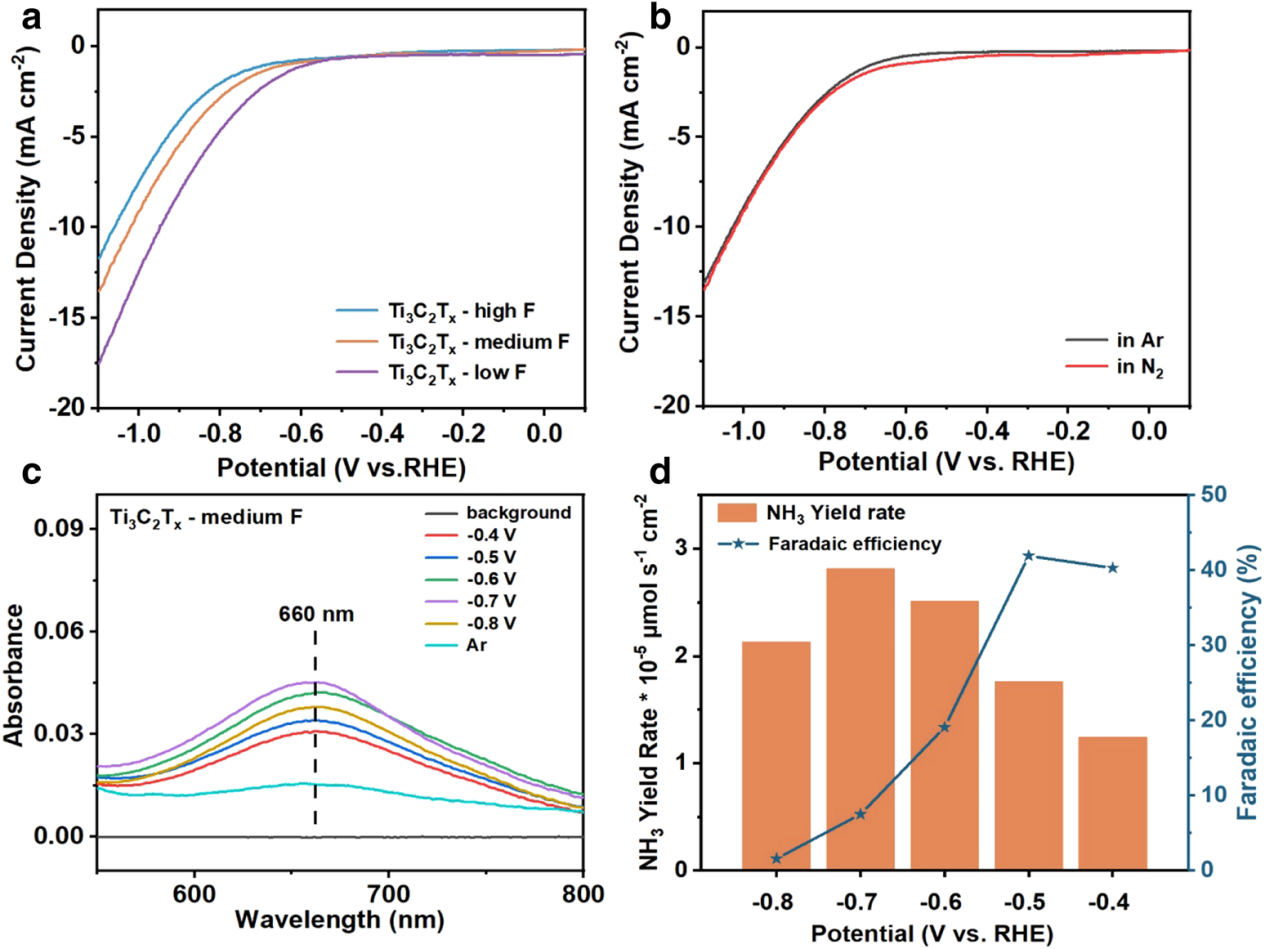
**a** Linear sweep voltametric curves of Ti_3_C_2_T_x_-low F, Ti_3_C_2_T_x_-medium F and Ti_3_C_2_T_x_-high F in N_2_-saturated 0.01 M Na_2_SO_4_ electrolyte with a scan rate of 5 mV·s^−1^. **b** LSV curves of Ti_3_C_2_T_x_-medium F in N_2_-saturated (red curve) and Ar-saturated (black curve) 0.01 M Na_2_SO_4_ electrolyte with a scan rate of 5 mV·s^−1^. **c** UV–Vis absorption spectra of N_2_RR products of Ti_3_C_2_T_x_-medium F at potentials between ‒0.4 and ‒0.8 V over Ti_3_C_2_T_x_ -48% F. **d** NH_3_ yield rate (left y-axis) and Faradaic efficiencies (right y-axis) of Ti_3_C_2_T_x_-medium F MXene at the corresponding potentials

The capability of different F-terminating surface functionalizations of Ti_3_C_2_T_x_ MXene for enhancing the N_2_RR catalytic activity was then investigated. All the electrochemical tests were conducted in N_2_-saturated 0.01 M Na_2_SO_4_ electrolyte, and all the potentials presented in this work were converted as values versus reversible hydrogen electrode (RHE). The linear sweep voltammetry (LSV) curves of Ti_3_C_2_T_x_ MXene with different proportions of fluorine terminal groups were recorded (Fig. 3a). The experimental overpotentials of Ti_3_C_2_T_x_ MXene displayed the following order of Ti_3_C_2_T_x_-high F > Ti_3_C_2_T_x_-medium F > Ti_3_C_2_T_x_-low F at ‒10 mA·cm^−2^ current density, which was associated with the hydrogenation step during electrochemical reduction. Owning to the different densities of F-termination on the MXene surface, the hydrogenation step could be inhibited [31]. The Ti_3_C_2_T_x_-high F sample had the highest density of fluorine terminal groups, resulting in the inhibition of H^+^ adsorption [32] and eventually the decline of N_2_RR activity. In contrast, Ti_3_C_2_T_x_-low F had a low density of fluorine terminal groups and abundant O-containing termination, and presented the highest hydrogen evolution reaction (HER) performance but low N_2_ adsorption and activation capabilities. In comparison, Ti_3_C_2_T_x_-medium F MXene had a medium density of fluorine terminal groups, and presented the highest N_2_RR electrocatalytic activity.

The Ti_3_C_2_T_x_-medium F MXene catalyst was further tested in both Ar-saturated and N_2_-saturated electrolytes (Fig. 3b). The current density in N_2_-saturated electrolyte (red curve) exceeded that in Ar-saturated electrolyte (black curve) in the voltage range between − 0.4 and − 0.8 V, indicating the occurrence of electrochemical N_2_RR on the catalyst surface. Both the chronoamperometric tests and the salicylic acid indicator method were adopted to determine the amount of produced NH_3_. All the yields of ammonia were calculated from the standard curves (Additional file 1: Fig. S6). In addition, each experiment was also conducted in Ar-saturated electrolyte to serve as the background. The corrected rate of NH_3_ yield (YR_corrected_) was calculated from the following equation: YR_corrected_ = YR_N2_ – YR_Ar_. The NH_3_ yield rate from Ti_3_C_2_T_x_-medium F MXene was calculated based on the corresponding UV–Vis absorption spectra at the potential range between ‒0.4 and ‒0.8 V (Fig. 3c). The value of average background (YR_Ar_) was calculated as (2.03 ± 0.2) × 10^–5^ μmol·s^−1^·cm^−2^. As shown in Fig. 3d, the maximum FE for NH_3_ production by Ti_3_C_2_T_x_-medium F MXene was 42.7% at ‒0.5 V, while the highest NH_3_ partial current density after background correction was 18.3 μA·cm^−2^ at ‒0.7 V, corresponding to an FE of 7.4% and the NH_3_ production rate of 2.81 × 10^–5^ μmol·s^−1^·cm^−2^.

**Fig. 4:**
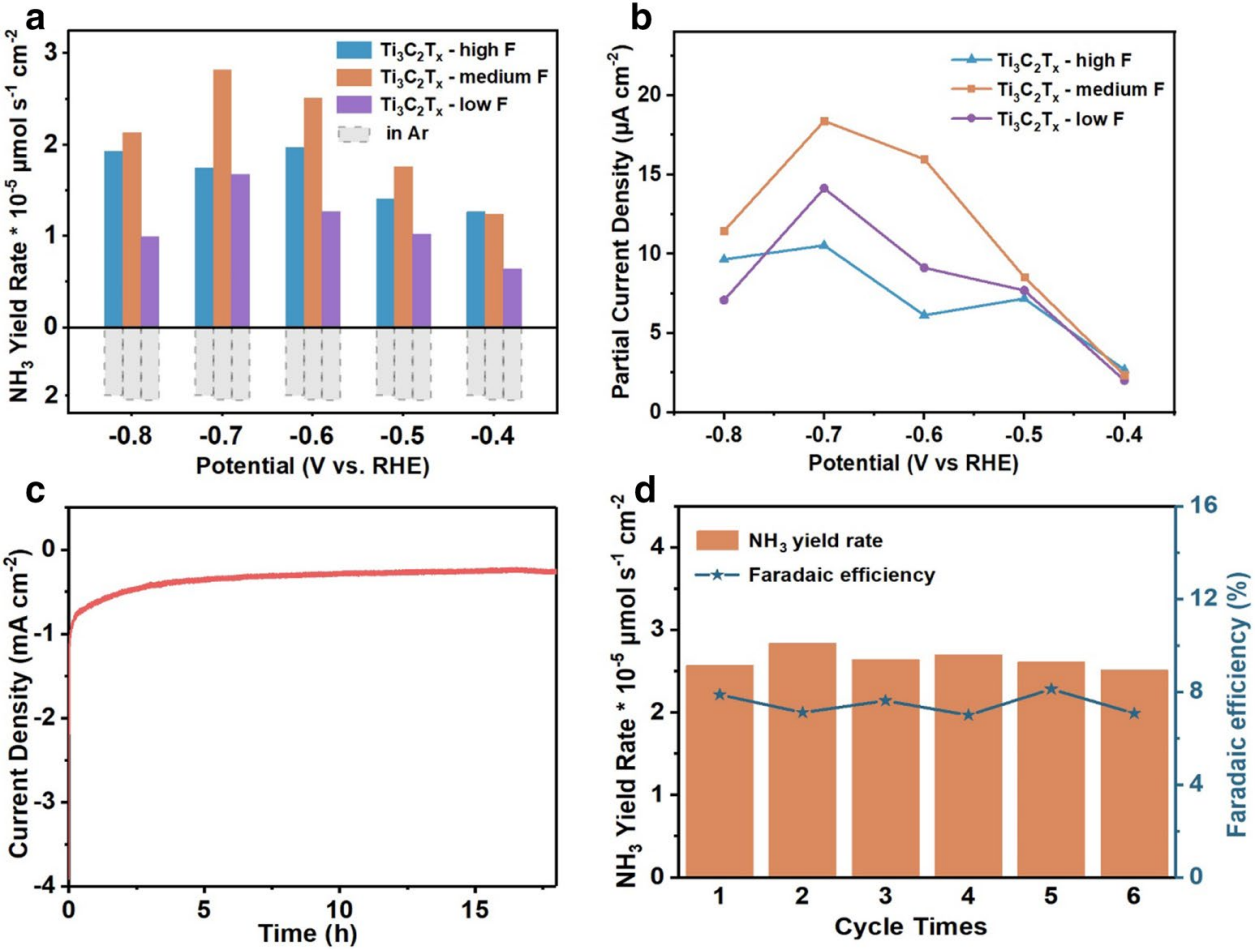
**a** The ammonia production rates and **b** partial current densities of NH_3_ production of Ti_3_C_2_T_x_ MXene samples with different surface densities of fluorine terminal groups. **c** Chronoamperometry curve of Ti_3_C_2_T_x_-medium F MXene for 18 h under ‒0.7 V. **d** The NH_3_ yield rate (left y-axis) and Faradaic efficiency (right y-axis) of Ti_3_C_2_T_x_-medium F MXene at ‒0.7 V for 6 times

The chronoamperometry curves and the corresponding UV–Vis absorption spectra over Ti_3_C_2_T_x_ MXene with different densities of F-terminal groups at all applied potentials were displayed (Additional file 1: Fig. S7). Compared to Ti_3_C_2_T_x_ MXene counterparts with higher or lower surface densities of fluorine terminal groups (i.e., Ti_3_C_2_T_x_-high F and Ti_3_C_2_T_x_-low F), the Ti_3_C_2_T_x_-medium F MXene catalyst covered with medium fluorine terminal group density exhibited the highest N_2_RR catalytic performance (Fig. 4a, b). The corrected NH_3_ yield rate (YR_corrected_) with Ti_3_C_2_T_x_-medium F catalyst (2.81 × 10^–5^ μmol·s^−1^·cm^−2^) was 1.6 and 1.7 times higher than that of Ti_3_C_2_T_x_-high F (1.75 × 10^–5^ μmol·s^−1^·cm^−2^) and Ti_3_C_2_T_x_-low F (1.67 × 10^–5^ μmol·s^−1^·cm^−2^) at ‒0.7 V.

The electrochemical stability of the Ti_3_C_2_T_x_-medium F MXene catalyst was further interrogated. As shown in Fig. 4c, the total electrolysis current density in N_2_-saturated electrolyte was maintained relatively stable over 18 h. Moreover, cycling test of six continuous times was conducted, and the corresponding chronoamperometric measurements and UV–Vis absorption spectra were examined after each cycle (Additional file 1: Fig. S7e, f). Both of NH_3_ production rate (2.67 ± 0.16 × 10^–5^ μmol·s^−1^·cm^−2^) and the FE values (7.5% ± 0.5%) were within the error range of 7.4% after the continuous chronoamperometric measurement for 6 times, with 1 h of measurement each time (Fig. 4d), which suggesting the excellent durability of Ti_3_C_2_T_x_-medium F MXene. Furthermore, the XRD patterns of Ti_3_C_2_T_x_ MXene before and after electrochemical nitrogen reduction reaction were also displayed (Additional file 1: Fig. S8), the unvaried peak position of XRD patterns also testified the stable crystal phases and structure of Ti_3_C_2_T_x_ MXene. Thus, Ti_3_C_2_T_x_-medium F MXene with a medium density of surface fluorine terminal groups was demonstrated as an optimal catalyst for N_2_RR.

## Conclusions

In summary, we demonstrated the surface functionalization of fluorine terminal groups on MXene to tune the N_2_RR catalytic activity at ambient conditions, in which different densities of surface fluorine terminal groups allowed to affect the capability of N_2_ adsorption and activation. The Ti_3_C_2_T_x_ MXene catalyst with a medium F-termination proportion (Ti_3_C_2_T_x_-medium F) showed the optimal N_2_RR activity, with the highest NH_3_ yield rate of 2.81 × 10^–5^ μmol·s^−1^·cm^−2^ at ‒0.7 V, substantially exceeding that of Ti_3_C_2_T_x_-high F and Ti_3_C_2_T_x_-low F. Further study and development of surface functionalization toward N_2_ adsorption and activation can serve as a powerful toolkit for improving artificial N_2_ fixation.

## Supplementary Information

**Additional file 1**: **Fig. S1**. (a-c) SEM images and (d) EDS elemental analysis profile of Ti3AlC2 MAX. **Fig. S2**. (a) The blackish green color and (b) the Tyndall scattering effect of Ti3C2Tx MXene solution. **Fig. S3**. SEM images of (a-c) Ti3C2Tx-high F; (d-f) Ti3C2Tx-medium F and (g-i) Ti3C2Tx-low F. **Fig. S4**. EDS elemental analysis profiles of (a) Ti3C2Tx-low F; (b) Ti3C2Tx-medium F (c) Ti3C2Tx-high F, and (d) the element analysis of all MXene samples. **Fig. S5**. (a) O 1s and (b) C 1s XPS spectra of Ti3C2Tx-low F, Ti3C2Tx-medium F and Ti3C2Tx-high F Mxene samples. **Fig. S6**. (a) UV-Vis absorption spectra of standard ammonia solutions with salicylic acid indicator. (b) Standard curves for determination of ammonia concentrations: y = 0.5408x ‒ 0.0035, R2 = 0.995. **Fig. S7**. (a, c) Chronoamperometry curves of N2RR in 0.01 M Na2SO4 solution at corresponding potentials: (a) Ti3C2Tx-high F and (c) Ti3C2Tx-low F. (b, d) UV-Vis absorption spectra of (b) Ti3C2Tx-high F and (d) Ti3C2Tx-low F after N2RR electrolysis at different potentials for 1 h. (e) Chronoamperometry curves and (f) UV-Vis absorption spectra of N2RR over Ti3C2Tx-medium F at the potential of −0.7 V for 6 times.** Fig. S8**. XRD patterns of Ti3C2Tx MXene (on carbon paper, CP) before and after electrochemical nitrogen reduction reaction at the potential of −0.7 V. **Table S1**. Comparison of the electrochemical N2RR performances for MXene-based catalysts.

## Data Availability

The datasets used and/or analyzed during the current study are available from the corresponding author on reasonable request.
