# Supplementary material for: Promoting N2 electroreduction to ammonia by fluorine-terminating Ti3C2Tx MXene
Source: Nano Converg. 2021 May 10;8:14. doi: 10.1186/s40580-021-00264-9 (PMC8107063; doi:10.1186/s40580-021-00264-9)
Supplement: Supplementary file 1 — Additional file 1: Fig. S1. (a-c) SEM images and (d) EDS elemental analysis profile of Ti3AlC2 MAX. Fig. S2. (a) The blackish green color and (b) the Tyndall scattering effect of Ti3C2Tx MXene solution. Fig. S3. SEM images of (a-c) Ti3C2Tx-high F; (d-f) Ti3C2Tx-medium F and (g-i) Ti3C2Tx-low F. Fig. S4. EDS elemental analysis profiles of (a) Ti3C2Tx-low F; (b) Ti3C2Tx-medium F (c) Ti3C2Tx-high F, and (d) the element analysis of all MXene samples. Fig. S5. (a) O 1s and (b) C 1s XPS spectra of Ti3C2Tx-low F, Ti3C2Tx-medium F and Ti3C2Tx-high F Mxene samples. Fig. S6. (a) UV-Vis absorption spectra of standard ammonia solutions with salicylic acid indicator. (b) Standard curves for determination of ammonia concentrations: y = 0.5408x ‒ 0.0035, R2 = 0.995. Fig. S7. (a, c) Chronoamperometry curves of N2RR in 0.01 M Na2SO4 solution at corresponding potentials: (a) Ti3C2Tx-high F and (c) Ti3C2Tx-low F. (b, d) UV-Vis absorption spectra of (b) Ti3C2Tx-high F and (d) Ti3C2Tx-low F after N2RR electrolysis at different potentials for 1 h. (e) Chronoamperometry curves and (f) UV-Vis absorption spectra of N2RR over Ti3C2Tx-medium F at the potential of −0.7 V for 6 times. Fig. S8. XRD patterns of Ti3C2Tx MXene (on carbon paper, CP) before and after electrochemical nitrogen reduction reaction at the potential of −0.7 V. Table S1. Comparison of the electrochemical N2RR performances for MXene-based catalysts. [file 40580_2021_264_MOESM1_ESM.docx]

Supporting Information

Promoting N_2_ electroreduction to ammonia by fluorine-terminating Ti_3_C_2_T_x_ MXene

Yu Ding^1^, Junbo Zhang^1^, Anxiang Guan^1^, Qihao Wang^1^, Si Li^1^, Abdullah M. Al-Enizi^2^, Linping Qian^1^, Lijuan Zhang^1,^*, Gengfeng Zheng^1,^*

^1^ Laboratory of Advanced Materials, Department of Chemistry, Faculty of Chemistry and Materials Science, Fudan University, Shanghai 200438, China.

^2^ Department of Chemistry, College of Science, King Saud University, Riyadh 11451, Saudi Arabia

*E-mail address: [gfzheng@fudan.edu.cn](mailto:gfzheng@fudan.edu.cn), zhanglijuan@fudan.edu.cn

**Supplementary Experimental Section**

*Preparation of working electrodes*: The concentrations of different MXene solutions were calibrated by a weighing method. In brief, 2 mL of MXene solution was dropped on a piece of carbon paper with an area of 0.5 cm^2^ (CP, 1 cm ⨯ 0.5 cm), and allowed the solvent to evaporate in an Ar environment to prepare the working electrode. The mass loading of Ti_3_C_2_T_x_ MXene was obtained via the weight difference between MXene/CP and CP, which was used to calibrate the concentrations of different Ti_3_C_2_T_x_ MXene solutions. In order to prevent catalyst falling off during the electrocatalytic process, 5 wt% Nafion ethyl alcohol dispersion was slightly added onto the catalyst side of carbon paper.

*Electrochemical measurements*: The electrochemical N_2_RR performances of all the aforementioned electrocatalysts were investigated in a three-electrode system by using a gas-tight H-type cell separated by the Nafion 117 membrane. An Ag/AgCl (in saturated KCl) reference electrode was used in the cathode compartment of the gas-tight H-type cell, and a platinum (Pt) counter electrode was used in the anode compartment. A 0.01 M aqueous Na_2_SO_4_ solution was used as the electrolyte. High purity gas was continuously delivered into the cathodic compartment at a constant flow rate during the experiment. The electrochemical data were collected on a CHI660D electrochemical workstation (CH Instrument Inc., USA), and all the potentials reported in the study were calibrated to the reversible hydrogen electrode (RHE) scale according to the formula: E_RHE_ = E + E_ref_ + 0.059 ⨯ pH, where E is the potential measured in all the N_2_RR tests, and E_ref_ is potential of the Ag/AgCl/3.5 M KCl reference electrode (which equals to 0.2046 V).

*Ammonia determination*: The concentrations of the produced NH_3_ were quantified by the salicylic acid indicator method. In brief, a color reagent was prepared by dissolving 5 g of salicylic acid, 5 g of potassium sodium tartrate tetrahydrate, and 8 g of sodium hydroxide in deionized (DI) water. Afterwards, 8 mL of the product solution was uniformly mixed with 1 mL of the aforementioned color reagent, 0.1 mL of sodium nitro-ferricyanide dehydrate solution, and additional DI water to make the total volume to 10 mL, and then the whole mixture was incubated at room temperature for 60 min. The formation of indophenol blue was determined using the absorbance at a wavelength of 660 nm by UV-Vis spectrophotometric measurements. The standard curves of ammonia were achieved using ammonium chloride solutions and updated every time before the production quantification.

*Indicators of ammonia production*: The calculations of ammonia production were described as follows. The actual mass of ammonia production (*m*) was calculated by the formula as $\text{m}\text{=}\frac{\left( \text{c}_{\text{N}}\text{ }\text{‒}{\text{ }\text{c}}_{\text{Ar}\text{ }}\text{‒ b} \right)}{\text{k}}\text{*V (μg)}$, where (*c*_N_ ‒ *c*_Ar_) indicates the actual concentration of produced ammonia calculated by the NH_3_ obtained in N_2_-saturated and Ar-saturated electrolytes; *k* and *b* are the slope and intercept of the standard calibration curves, respectively; *V* is the volume of the solution. The Faradaic efficiency (FE) was calculated by: $\text{FE=}\frac{\text{m}\text{ }\text{*}\text{ }\text{3}\text{ }\text{*}\text{ }\text{F}}{\text{17Q}}\text{*}\text{10}^{\text{-6}}$, where *F* is Faraday constant (96485.34 C·mol^-1^), *Q* is the total charge passing through the electrochemical system. The NH_3_ yield rate (YR) was calculated by the formula as: $\text{YR=}\frac{\text{m}}{\text{t}\text{ }\text{*}\text{ }\text{A}\text{ }\text{*}\text{ }\text{17}}\text{ (}\text{μ}\text{mol }\text{s}^{\text{-1}}\text{ }\text{cm}^{\text{-2}}\text{)}$, where *t* is the reaction time, *A* is the electrode area of catalysts. The partial current density (P*j*) was calculated by$P_{j}\text{ }\text{=}\text{ }\text{j}\text{ }\text{*}\text{ }\text{FE}\text{ }\text{*}\text{ }\text{10}^{\text{3}}\text{ }\left( \text{μA }\text{cm}^{\text{-2}} \right)$, where *j* is the total current density of the chronoamperometry curves at different working potentials.

**Supplementary Figures**


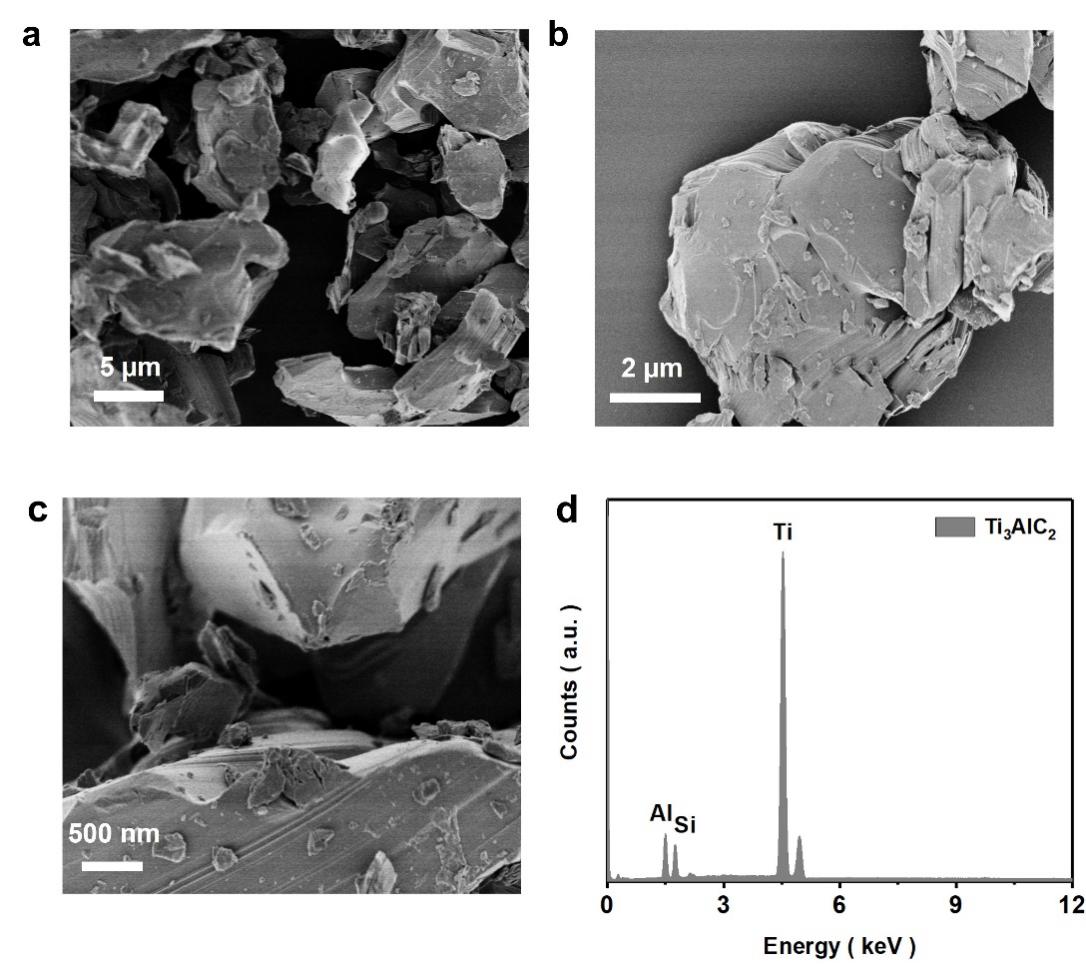


**Fig. S1.** (a-c) SEM images and (d) EDS elemental analysis profile of Ti_3_AlC_2_ MAX.


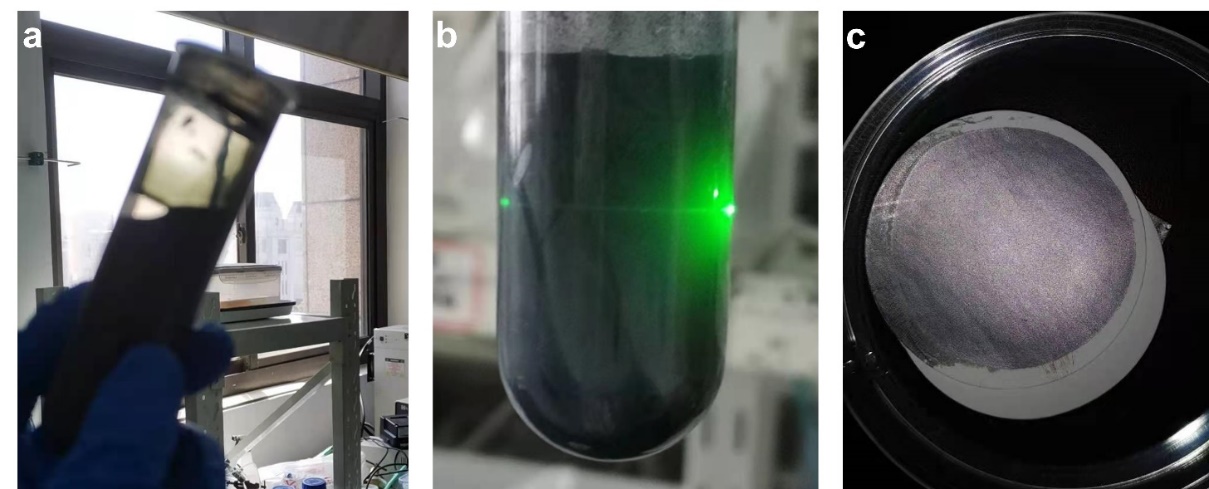


**Fig. S2.** (a) The blackish green color and (b) the Tyndall scattering effect of Ti_3_C_2_T_x_ MXene solution.


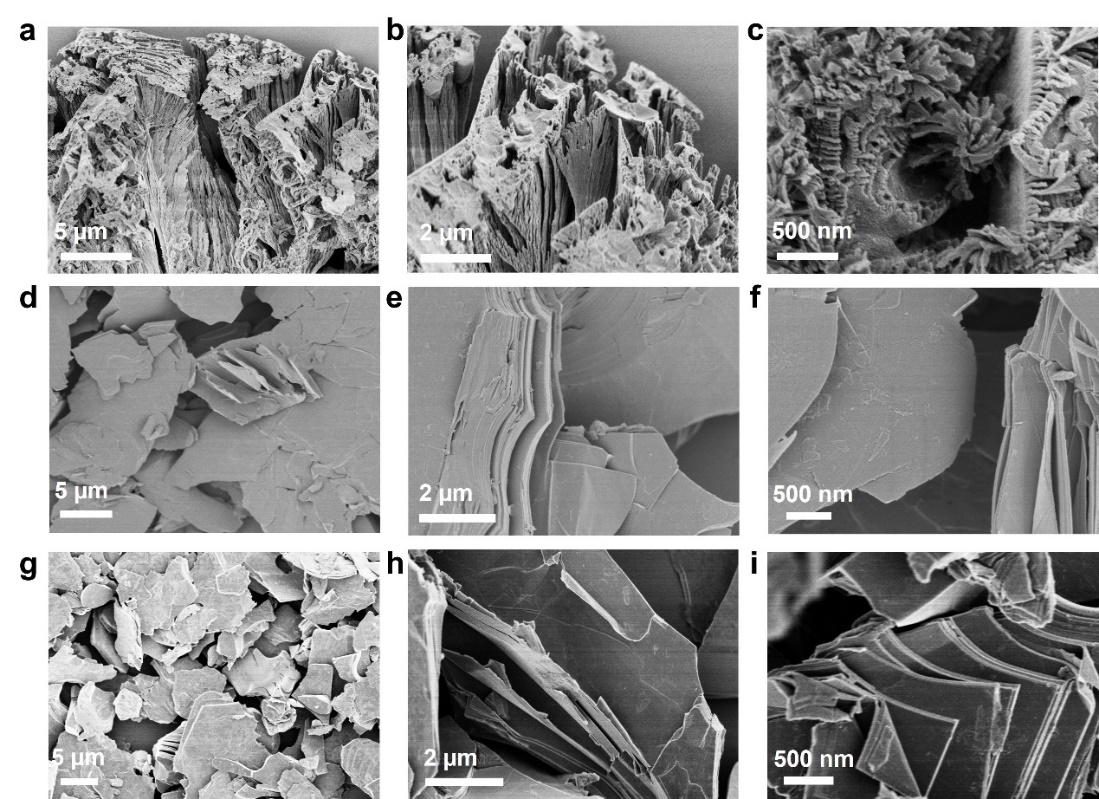


**Fig. S3.** SEM images of (a-c) Ti_3_C_2_T_x_-high F; (d-f) Ti_3_C_2_T_x_-medium F and (g-i) Ti_3_C_2_T_x_-low F.


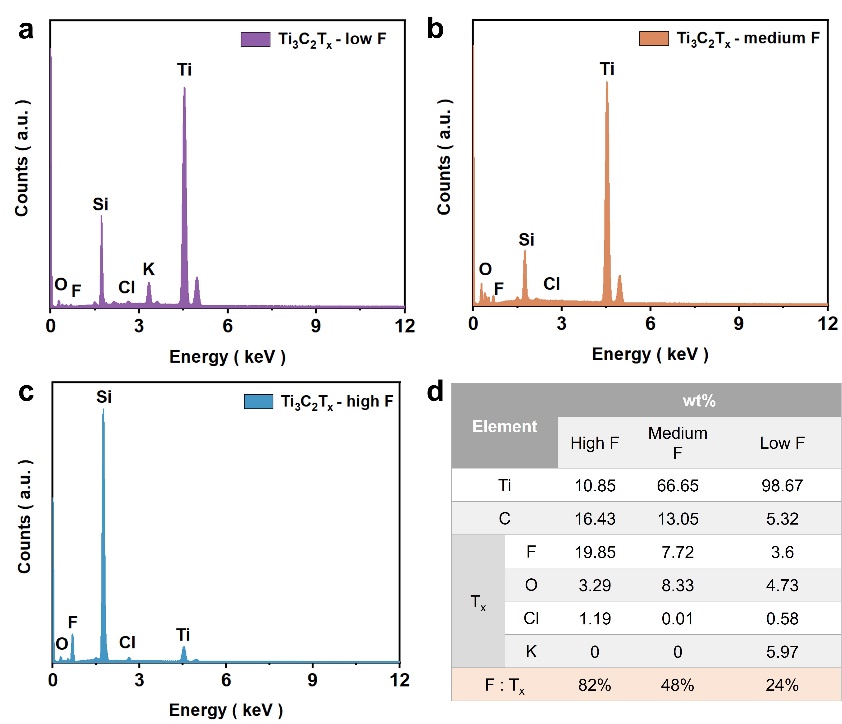


**Fig. S4.** EDS elemental analysis profiles of (a) Ti_3_C_2_T_x_-low F; (b) Ti_3_C_2_T_x_-medium F (c) Ti_3_C_2_T_x_-high F, and (d) the element analysis of all MXene samples.


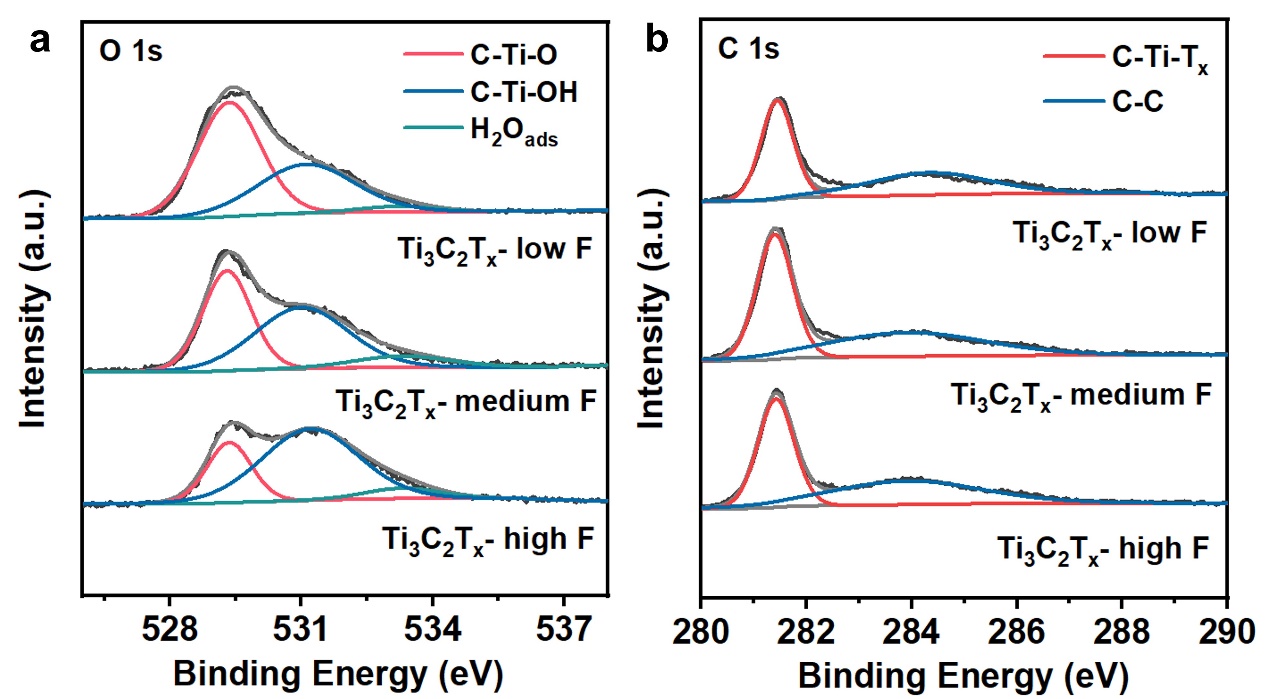


**Fig. S5.** (a) O 1s and (b) C 1s XPS spectra of Ti_3_C_2_T_x_-low F, Ti_3_C_2_T_x_-medium F and Ti_3_C_2_T_x_-high F Mxene samples.


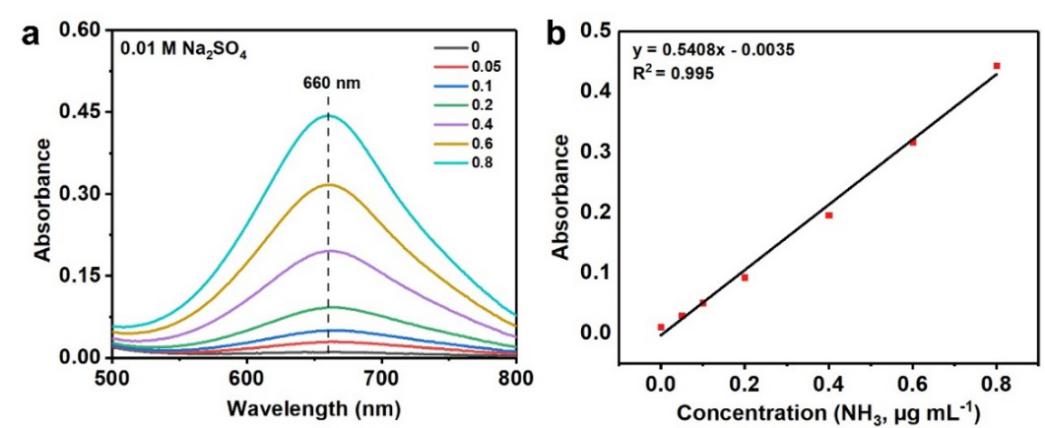


**Fig. S6.** (a) UV-Vis absorption spectra of standard ammonia solutions with salicylic acid indicator. (b) Standard curves for determination of ammonia concentrations: *y* = 0.5408*x* ‒ 0.0035, *R*^2^ = 0.995.


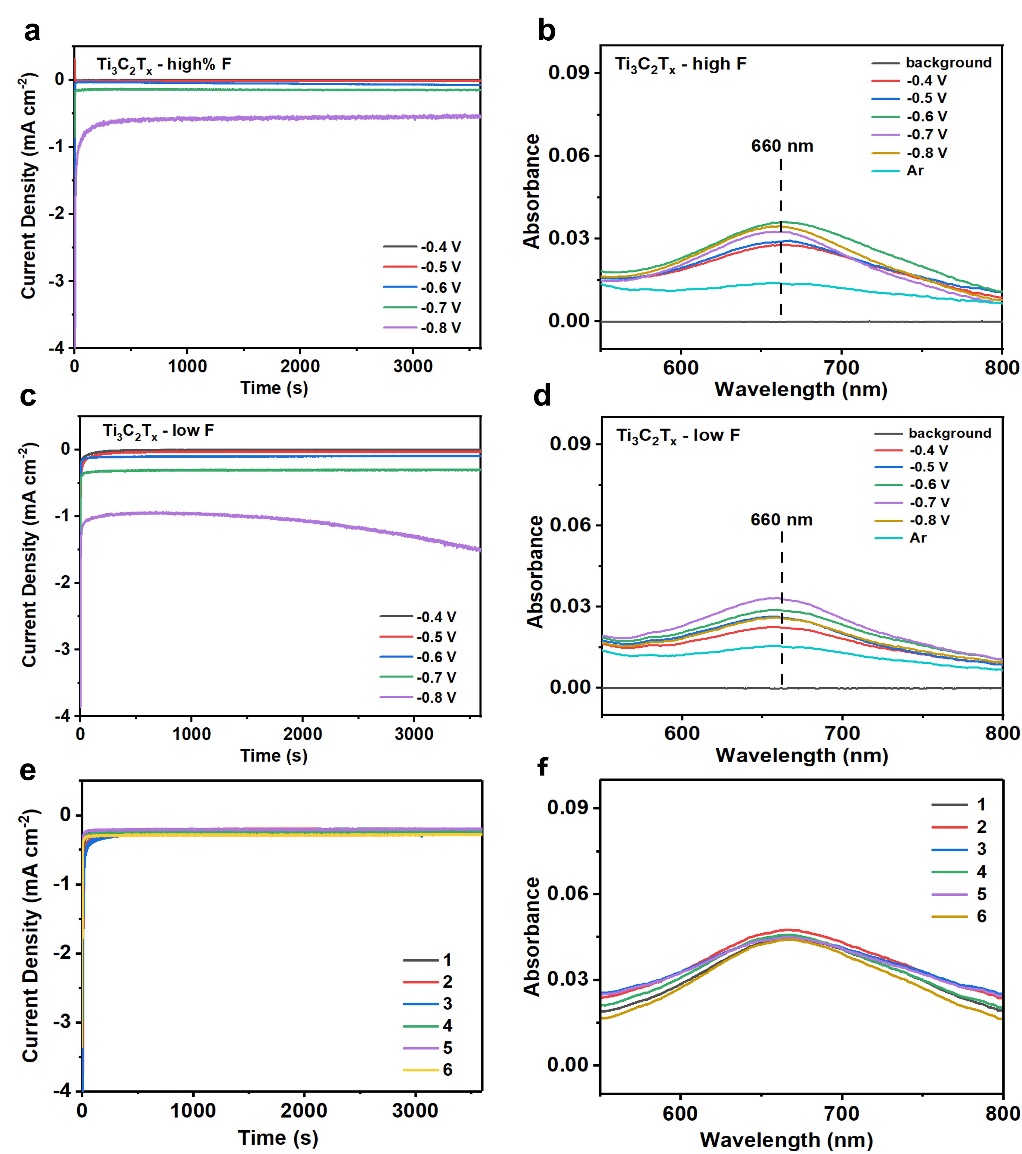


**Fig. S7.** (a, c) Chronoamperometry curves of N_2_RR in 0.01 M Na_2_SO_4_ solution at corresponding potentials: (a) Ti_3_C_2_T_x_-high F and (c) Ti_3_C_2_T_x_-low F. (b, d) UV-Vis absorption spectra of (b) Ti_3_C_2_T_x_-high F and (d) Ti_3_C_2_T_x_-low F after N_2_RR electrolysis at different potentials for 1 h. (e) Chronoamperometry curves and (f) UV-Vis absorption spectra of N_2_RR over Ti_3_C_2_T_x_-medium F at the potential of −0.7 V for 6 times.


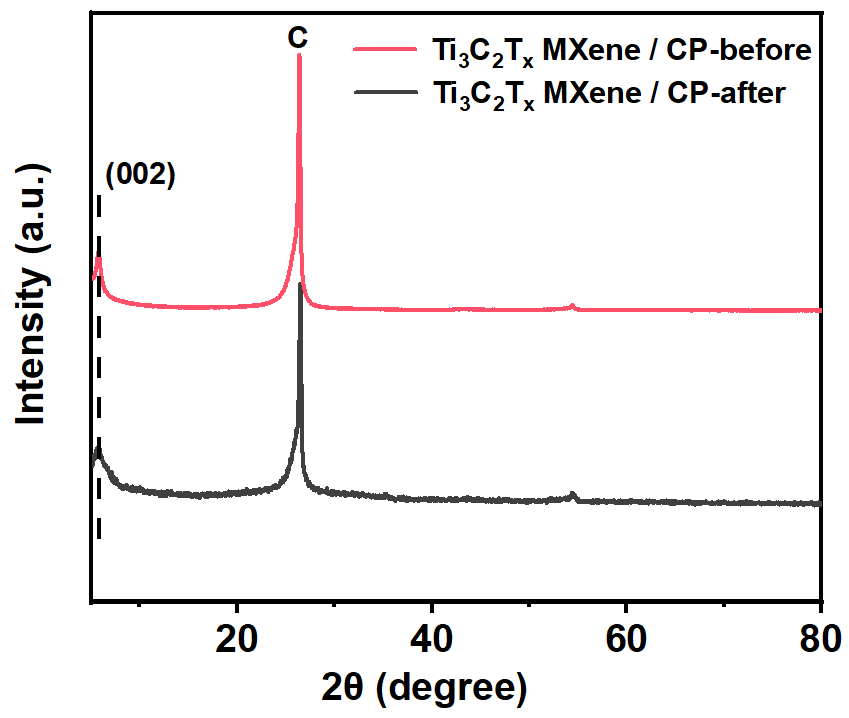


**Fig. S8.** XRD patterns of Ti_3_C_2_T_x_ MXene (on carbon paper, CP) before and after electrochemical nitrogen reduction reaction at the potential of −0.7 V.

**Table S1.** Comparison of the electrochemical N_2_RR performances for MXene-based catalysts.

| **Catalyst** | **NH_3_ Yield Rate** | **FE (%)** | **Electrolyte** | **Ref.** |
| --- | --- | --- | --- | --- |
| Ti_3_C_2_T_x_-medium F | 2.81 ×10^-5^ μmol s^-1^ cm^-2^  (or: 1.72 μg h^-1^ cm^-2^) | 7.38 | 0.01 M Na_2_SO_4_ | This work |
| MXene/SSM | 4.72 μg h^-1^ cm^-2^ | 4.62 | 0.01 M HCl | Ref.^S1^ |
| Ti_3_C_2_OH | 1.71 μg h^-1^ cm^-2^ | 7.01 | 0.1 M KOH | Ref. ^S2^ |
| MXene/TiFeO_x_-700 | 2.19 μg h^-1^ cm^-2^ | 25.44 | 0.05 M H_2_SO_4_ | Ref. ^S3^ |
| Cu/Ti_3_C_2_ | 3.04 μg h^-1^ cm^-2^ | 7.31 | 0.1 M KOH | Ref. ^S4^ |
| Ti_3_C_2_OH QDs | 62.94 µg h^-1^ mg^-1^ | 13.30 | 0.1 M HCl | Ref. ^S5^ |
| Ti_3_C_2_T_x_ (T = O, OH) | 36.9 μg h^-1^ mg^-1^ | 9.1 | 0.1 M HCl | Ref. ^S6^ |
| TiO_2_/Ti_3_C_2_T_x_ | 26.32 μg h^-1^ mg^-1^ | 8.42 | 0.1 M HCl | Ref. ^S7^ |
| 1T-MoS_2_@Ti_3_C_2_ | 30.33 μg h^−1^ mg^−1^ | 10.94 | 0.1 M HCl | Ref. ^S8^ |
| Single-atomic Ru-Mo_2_CT_X_ | 40.57 μg h^−1^ mg_._^−1^ | 25.77 | 0.5 M K_2_SO_4_ | Ref. ^S9^ |

**References**

[S1]. Luo, Y.; Chen, G.-F.; Ding, L.; Chen, X.; Ding, L.-X.; Wang, H., Efficient electrocatalytic N_2_ fixation with MXene under ambient conditions. *Joule* **2019,** *3* (1), 279-289.

[S2]. Xia, J.; Yang, S.-Z.; Wang, B.; Wu, P.; Popovs, I.; Li, H.; Irle, S.; Dai, S.; Zhu, H., Boosting electrosynthesis of ammonia on surface-engineered MXene Ti_3_C_2_. *Nano Energy* **2020,** *72*, 104681.

[S3]. Guo, Y.; Wang, T.; Yang, Q.; Li, X.; Li, H.; Wang, Y.; Jiao, T.; Huang, Z.; Dong, B.; Zhang, W.; Fan, J.; Zhi, C., Highly efficient electrochemical reduction of nitrogen to ammonia on surface termination modified Ti_3_C_2_T_x_ MXene nanosheets. *ACS Nano* **2020,** *14* (7), 9089-9097.

[S4]. Liu, A.; Liang, X.; Yang, Q.; Ren, X.; Gao, M.; Yang, Y.; Ma, T., Electrocatalytic synthesis of smmonia using a 2D Ti_3_C_2_ MXene loaded with copper nanoparticles. *Chempluschem.* **2021,** *86* (1), 166-170.

[S5]. Jin, Z.; Liu, C.; Liu, Z.; Han, J.; Fang, Y.; Han, Y.; Niu, Y.; Wu, Y.; Sun, C.; Xu, Y., Rational design of hydroxyl‐rich Ti_3_C_2_T_x_ MXene quantum dots for high‐performance electrochemical N_2_ reduction. *Advanced Energy Materials* **2020,** *10* (22), 45-56.

[S6]. Li, T.; Yan, X.; Huang, L.; Li, J.; Yao, L.; Zhu, Q.; Wang, W.; Abbas, W.; Naz, R.; Gu, J.; Liu, Q.; Zhang, W.; Zhang, D., Fluorine-free Ti_3_C_2_T_x_ (T = O, OH) nanosheets (∼50–100 nm) for nitrogen fixation under ambient conditions. *Journal of Materials Chemistry A* **2019,** *7* (24), 14462-14465.

[S7]. Zhang, J.; Yang, L.; Wang, H.; Zhu, G.; Wen, H.; Feng, H.; Sun, X.; Guan, X.; Wen, J.; Yao, Y., In-Situ hydrothermal growth of TiO_2_ nanoparticles on a conductive Ti_3_C_2_Tx MXene nanosheet: A synergistically active Ti-based nanohybrid electrocatalyst for enhanced N_2_ reduction to NH_3_ at ambient conditions. *Inorg. Chem.* **2019,** *58* (9), 5414-5418.

[S8]. Xu, X.; Sun, B.; Liang, Z.; Cui, H.; Tian, J., High-performance electrocatalytic conversion of N_2_ to NH_3_ using 1T-MoS_2_ anchored on Ti_3_C_2_ MXene under ambient conditions. *ACS Appl. Mater. Interfaces* **2020,** *12* (23), 26060-26067.

[S9]. Peng, W.; Luo, M.; Xu, X.; Jiang, K.; Peng, M.; Chen, D.; Chan, T. S.; Tan, Y., Spontaneous atomic ruthenium doping in Mo_2_CT_X_ MXene defects enhances electrocatalytic activity for the nitrogen reduction reaction. *Advanced Energy Materials* **2020,** *10* (25), 2001364.
